# The Country Connector on Private Sector in Health: a global governance platform

**DOI:** 10.1136/bmjgh-2023-014454

**Published:** 2023-12-27

**Authors:** David Clarke, Anna Cocozza, Ernesto Bascolo, Hassan Salah, Gabriele Pastorino, Muriel Ngum Anye, Omar Sam

**Affiliations:** 1 Special Programme on Primary Health Care, World Health Organization, Geneva, Switzerland; 2 Pan American Health Organization, Washington, DC, USA; 3 World Health Organisation Regional Office for the Eastern Mediterranean, Cairo, Egypt; 4 World Health Organization Regional Office for Europe, Copenhagen, Denmark; 5 World Health Organization Regional Office for Africa, Brazzaville, Congo

**Keywords:** Health policies and all other topics, Health policy, Health systems

**Summary box**

- The pursuit of UHC faces challenges exacerbated by the COVID-19 pandemic, necessitating comprehensive approaches involving both public and private health stakeholders.
- Launched by the World Health Organization (WHO), the Country Connector on private sector in health (CCPSH) serves as a global platform to foster collaboration, share experiences, and provide guidance to strengthen mixed health systems, aligning efforts of public and private sectors towards UHC and health security.
- Pioneering initiatives like HANSHEP (2010) laid the foundation for enhancing the performance of the non-state sector in delivering improved healthcare to underserved populations. The emergence of the COVID-19 pandemic prompted the creation of the CCPSH, emphasizing the importance of all stakeholders’ efforts, both public and private, in pursuing UHC.
- By fostering synergy across boundaries and sectors, the Country Connector aims to create a healthcare ecosystem that ensures equitable and comprehensive healthcare for all, leaving no one behind on the journey towards UHC.

## Introduction

The world faces a pivotal challenge in pursuing universal health coverage (UHC). The global community acknowledges that progress towards UHC has faltered, and the devastating impact of the COVID-19 pandemic has pushed us further away from the targets set by the 2019 political declaration on UHC.^1^ The 2023 political declaration of the high-level meeting on UHC reiterated the vital role of political leadership in adopting comprehensive government and societal approaches.^2^ As other articles in this Supplement pointed out, progressing towards UHC calls for the commitment of all health stakeholders, both public and private.

Governments play a central role of stewardship in governing all health actors, transitioning from service providers to systems ‘steerers’.^3^ This transformation requires policy instruments and institutional capacity for effective health systems reforms that include private actors. Recent years have seen increased efforts from global, regional and national actors to assist countries in working with the private sector in healthcare, yielding valuable insights and best practices. In 2021, this knowledge laid the foundation for launching a global platform to enhance worldwide co-ordination of programmes and activities dedicated to strengthening mixed health systems—where both public and private actors provide health services—including engagement with the private sector in health.

Convened by the WHO, the Country Connector on Private Sector in Health (CCPSH) was launched in response to the demand for support on how to work with the private sector towards achieving UHC. The CCPSH serves as a platform for sharing experiences, establishing norms, supporting learnings and providing the guidance needed for stronger governance of mixed health systems to ensure everyone has equal access to quality essential health services, preventing financial hardships. This commentary sheds light on the establishment and significance of the CCPSH, as a platform to support countries fostering a whole health system approach to strengthening health systems, aligning the efforts of both the public and private sectors towards the common objectives of UHC and health security.

## Pioneering initiatives and global actions

In 2009, the Taskforce for Innovative International Financing for Health Systems released a ground-breaking report that explored key factors vital for achieving the health Millennium Development Goals (Sustainable Development Goals, SDGs) by 2015.^4^ This report highlighted the necessity of managing, harnessing, mobilising and enhancing the performance of the non-state sector alongside strengthening the government’s role in governance, regulation, contracting and quality improvement in healthcare. It underscored the importance of this often-neglected aspect in the healthcare domain. Consequently, the Taskforce recommended strengthening governments' capacity to engage with private, faith-based, community, NGO and other non-state actors in the health sector.

In response to this recommendation, 2010 marked the establishment of a new global initiative, the ‘Harnessing Non-State Actors for Better Health for the Poor’ platform (HANSHEP). HANSHEP served as a collaborative platform, bringing together development agencies, governments and foundations with a shared interest in supporting pro-poor mixed health systems. Its primary objective was to facilitate knowledge sharing and cofinancing of new initiatives. HANSHEP was founded by its members in 2010 and focused on enhancing the performance of the non-state sector in delivering improved healthcare to underserved populations. It fostered collaboration, mutual learning and the dissemination of knowledge to the broader community.

HANSHEP provided a space for members to collaboratively design, finance and scale programmes with shared objectives, including enhancing the delivery of quality healthcare to impoverished populations; reducing out-of-pocket expenditures for medicines and treatment among the poor; expanding access to quality health systems for marginalised communities; lowering the cost of medicines; increasing access to information on preventive care, treatment options and fair pricing for services; improving access to affordable priority disease-specific interventions; and strengthening financial protection for the poor against catastrophic healthcare expenses.

The year 2015 marked a notably significant milestone with the launch of the 2030 SDGs. SDG 17 called explicitly for co-operation, collaboration and partnership between government, civil society and businesses to achieve the agenda’s goals.^5^ In the health sector, this necessitated effective harnessing and steering of the public and private sectors to achieve health-related targets, notably SDG target 3.8 on UHC. This target encompassed financial risk protection, access to quality essential healthcare services and access to safe, effective, quality and affordable essential medicines and vaccines for all.

## WHO’s work on the governance of the private sector in health

Building on the emergent evidence of the need to govern both public and private actors to deliver health goals, in 2010, the WHO^6^ endorsed the World Health Assembly resolution WHA63.27 on ‘strengthening the capacity of governments to constructively engage the private sector in providing essential healthcare services’.^7^ This endorsement underscored the global recognition of the private sector’s role in healthcare delivery and the need for effective governance mechanisms to ensure its alignment with public health objectives. To align with SDG 2030, Members States within the WHO’s Eastern Mediterranean Region adopted resolution EM/RC65/R.3 in October 2018, endorsing a framework for effective engagement with the private health sector. The resolution urges Member States to integrate such engagement into their national policies and strengthen ministries of health to manage and evaluate private sector involvement in health service delivery.

The emergence of the COVID-19 pandemic in 2020 served as a stark reminder of the critical importance of engaging all stakeholders to respond to the pandemic. In the wake of this crisis, the WHO emphasised the importance of engaging all stakeholders, including private and public health actors, in pursuing UHC. This is to build resilient health systems capable of addressing the immediate challenges posed by pandemics and the long-term goal of achieving UHC for all. The same year, the WHO convened an Advisory Group on the Governance of the Private Sector for Universal Health Coverage. This Advisory Group developed a comprehensive strategy report titled ‘Engaging the private health service delivery sector through governance in mixed health systems’.^8^ This strategy report informed the design and conduct of the African Union–WHO multicountry study on the status of private sector engagement, opportunities available and strategic directions using the mix health approach.^9^

The strategy notably highlighted the need to align global, regional and local partners working in this domain and to bolster governance capacity at the country level to work effectively with the private sector in health. While providing a growing number of evidence and information on the governance of the private sector in health,^10^ in 2021, WHO launched the CCPSH as a platform to support countries engaging with the private sector to address the COVID-19 pandemic and achieve public health goals.

At the same time, the HANSHEP partners decided to conduct a strategic review of the platform to support the setup of the CCPSH and the transition of HANSHEPs operations to this new platform.

The primary objectives of the CCPSH have been to facilitate co-ordination efforts among multiple actors, including the private sector, in delivering critical healthcare interventions, such as COVID-19 vaccines, therapeutics and diagnostics. During the pandemic, it played a crucial role in providing countries with tools and support to effectively harness the private sector in health to respond to emergencies and build more resilient and well-prepared health systems. Gains made during the pandemic need to be reinforced and sustained. Key components of the CCPSH included: (1) working groups that brought together country actors, technical experts and development partners to tackle challenges on the governance of the private sector in health; (2) a clearing house to search for relevant information in the literature; (3) a tool repository, to offer practical guidance that supports action-oriented interventions; (4) a research and learning channel responsible for curating evidence and capturing insights from regional and country experiences; (5) a training course designed to help build the skills and knowledge necessary for effectively engaging the private sector in health; and (6) a support desk that worked across all three levels of WHO and with diverse stakeholders to provide tailored assistance in addressing governance challenges.

The success of the activities undertaken by the CCPSH prompted the WHO Secretariat to revamp the platform, intending to extend support to countries beyond the COVID-19 pandemic. With the goal of implementing recommendations from the WHO’s strategy report, the CCPSH now aims to facilitate knowledge sharing among countries. It connects them with essential resources, tools and guidance to enhance health system governance and inform more effective public policies regarding the private sector’s involvement in healthcare.

The CCPSH’s multifaceted approach encompasses curating evidence, enhancing advocacy, informing policies, supporting implementation and sharing experiences. In this context, it represents a collaborative effort involving global, regional and local actors committed to establishing an extensive knowledge hub focused on private sector governance in healthcare. This initiative involves carefully constructing a rich repository of evidence, insights and invaluable insights to guide policy development and implementation. Through its specialised country support desk, the CCPSH also provides tailored assistance to countries interested in engaging with the private sector in healthcare. Collaboration forms the core of the CCPSH’s mission, as it actively fosters partnerships with a diverse range of experts and stakeholders through the Private Sector Engagement Forum and the Technical Working Groups. These collaborative efforts facilitate the exchange of knowledge and experiences, driving meaningful progress in healthcare governance.

The CCPSH accomplishes the idea that to achieve UHC, a concerted effort is required on a global, regional and local scale, emphasising collaboration and co-operation among all stakeholders involved in the global health arena. Members of the CCPSH are from UN agencies, international organisations and private sector. By fostering synergy across boundaries and sectors, the CCPSH aims to create a healthcare ecosystem that ensures equitable and comprehensive healthcare for all, leaving no one behind on the journey towards UHC.

## Conclusion

The journey from realising the untapped potential of non-state actors in healthcare to creating platforms like HANSHEP and the CCPSH reflects a global commitment to making healthcare better and more accessible. These initiatives highlight the importance of implementing effective governance in healthcare systems where both public and private sectors coexist. Our shared goals of achieving UHC and adapting to the changing healthcare landscape stay front and centre as we move forward. The lessons we have learnt from these initiatives and the dedication of CCPSH members show us the way forward for stronger health systems where everyone can access high-quality healthcare without barriers.

## Data Availability

There are no data in this work.
